# The mitoXplorer 2.0 update: integrating and interpreting mitochondrial expression dynamics within a cellular context

**DOI:** 10.1093/nar/gkac306

**Published:** 2022-05-07

**Authors:** Fabio Marchiano, Margaux Haering, Bianca Hermine Habermann

**Affiliations:** Aix-Marseille University, CNRS, IBDM UMR 7288, 13009 Marseille, France; Aix-Marseille University, CNRS, IBDM UMR 7288, 13009 Marseille, France; Aix-Marseille University, CNRS, IBDM UMR 7288, 13009 Marseille, France

## Abstract

Mitochondria are subcellular organelles present in almost all eukaryotic cells, which play a central role in cellular metabolism. Different tissues, health and age conditions are characterized by a difference in mitochondrial structure and composition. The visual data mining platform mitoXplorer 1.0 was developed to explore the expression dynamics of genes associated with mitochondrial functions that could help explain these differences. It, however, lacked functions aimed at integrating mitochondria in the cellular context and thus identifying regulators that help mitochondria adapt to cellular needs. To fill this gap, we upgraded the mitoXplorer platform to version 2.0 (mitoXplorer 2.0). In this upgrade, we implemented two novel integrative functions, network analysis and transcription factor enrichment, to specifically help identify signalling or transcriptional regulators of mitochondrial processes. In addition, we implemented several other novel functions to allow the platform to go beyond simple data visualization, such as an enrichment function for mitochondrial processes, a function to explore time-series data, the possibility to compare datasets across species and an IDconverter to help facilitate data upload. We demonstrate the usefulness of these functions in three specific use cases. mitoXplorer 2.0 is freely available without login at http://mitoxplorer2.ibdm.univ-mrs.fr.

## Graphical Abstract

**Graphical Abstract:**
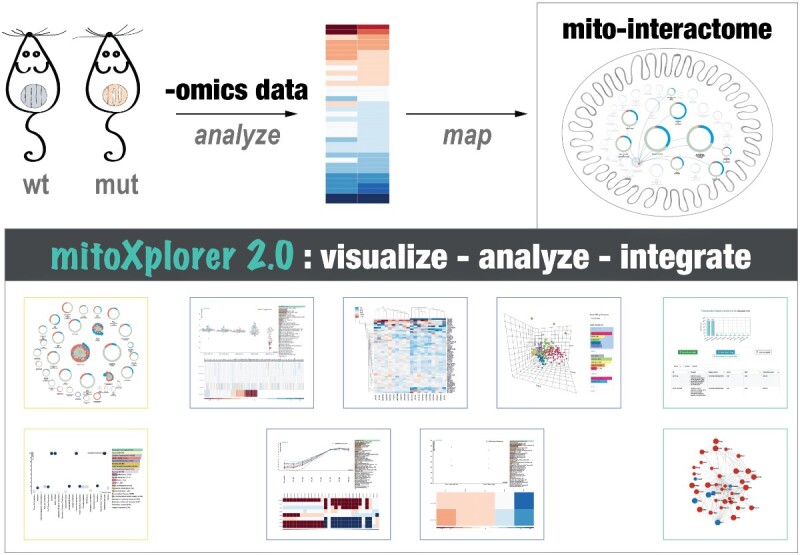
Mitochondria change their structure, function and content depending on the cell type and condition they are in. But how do they adapt to their cellular environment? The web platform mitoXplorer 2.0 allows us to visualize, analyse and integrate -omics data from a mitochondrial perspective. Differential expression data are mapped to the most up-to-date and complete mitochondrial interactomes available for four species. Seven visualization and analysis interfaces allow us to explore mitochondrial expression dynamics. Two new integration functions allow us to integrate data and help find transcriptional as well as signalling regulators of mitochondrial functions and thus help identify potential mechanisms of mitochondrial adaptation.

## INTRODUCTION

Mitochondria are essential organelles in most eukaryotic cells and are involved in a multitude of cellular processes, including cellular energy production, metabolism, signalling or apoptosis. The mitochondrial protein content is estimated to be around 1000 proteins, varying slightly between species (1–3). Most of these proteins (mito-proteins) are encoded in the nucleus and imported into mitochondria in a controlled manner. Only few mito-proteins are encoded in the mitochondrial genome and transcribed and translated within mitochondria, encoding proteins of the respiratory chain, ribosomal RNAs required for the mitochondrial ribosome and transfer RNAs. Mitochondria are essential for the functioning of the cell; however, the requirements on mitochondria vary with the cell type. Consequently, mitochondrial structure and protein content differ between cell types (4) and we want to understand how mitochondria adapt to their cellular environment.

mitoXplorer version 1.0 (mitoXplorer 1.0) was developed as a visual data mining platform that allows us to analyse the dynamics of gene expression and mutations of all genes with a mitochondrial function (mito-genes) (1). It contains the most complete and up-to-date mitochondrial interactomes for four different model species (human, mouse, *Drosophila melanogaster* and budding yeast) as well as four different visualization interfaces that enable users to mine expression and mutation data in a comparative manner, as well as from a single dataset. mitoXplorer 1.0 was the first platform available to mine expression dynamics of mito-genes in different conditions. However, it fails to integrate the mitochondrial interactome into the cellular context and thus to help identify potential signalling and transcriptional regulators that adjust mitochondria and their functions to their cellular environment.

We here introduce mitoXplorer version 2.0 (mitoXplorer 2.0), in which we have added integrative data analysis functions to help address these questions. MitoXplorer 2.0 contains several new functions for data analysis, such as an enrichment function for mito-processes, a function to explore time-series data, the possibility to compare datasets across species and two novel data integration functions that aim at identifying mitochondrial regulators: first, a function to identify potential transcriptional regulators of mito-genes with a similar expression profile (co-regulated mito-genes) by making use of our recently published AnnoMiner web tool (5); and second, a function to identify potential signalling pathways from and to mitochondria, by embedding the mito-interactome in the cellular interactome and subsequently exploring the network neighbourhood of a selected mito-gene based on integrating the network with differential expression data (6). We demonstrate the usefulness of these new functions in three use cases, where we identify potential transcriptional regulators driving the mitochondrial metabolic switch during *Drosophila* flight muscle development (use case 1, Figure 2), identify potential active signalling pathways regulating Ca^2+^ signalling in ataxia (use case 2, Figure 3) and explore conserved mito-gene deregulation in fibroblasts from human and a mouse model of trisomy 21 (use case 3, Supplementary Figure S1).

## MATERIALS AND METHODS

**Figure 1.**
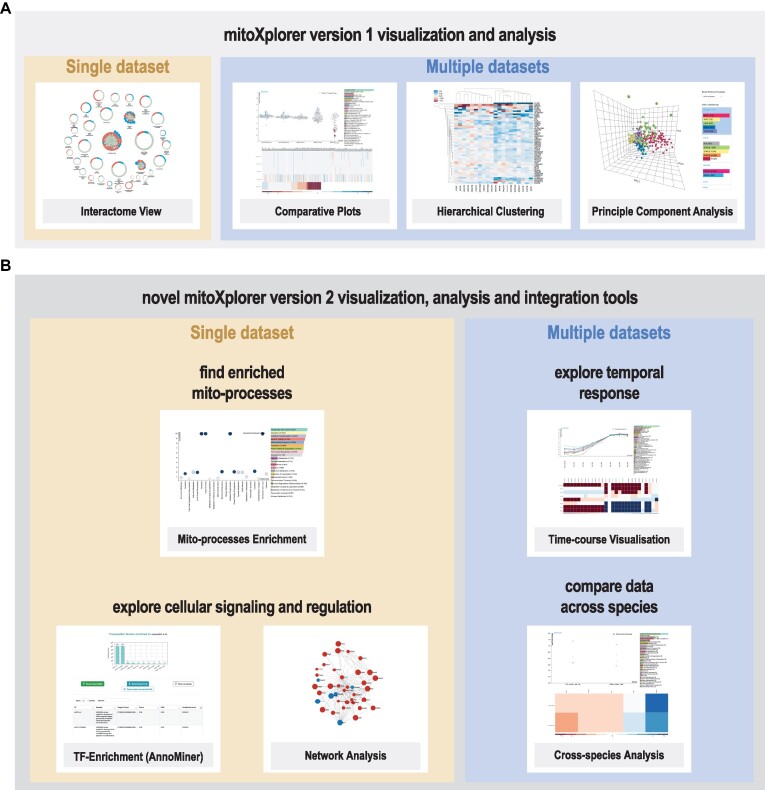
Collection of functions available in mitoXplorer 2.0. **(A)** Visualization and analysis tools available already in mitoXplorer 1.0 include the interactome view, comparative plots, hierarchical clustering and principal component analysis. **(B)** Novel functions available in mitoXplorer 2.0 are enrichment of mito-processes, time-course visualization for up to 20 time points, cross-species analysis, as well as the data integration functions of transcription factor (TF)-enrichment via AnnoMiner and network analysis using the viPEr algorithm.

Novel functions of mitoXplorer version 2.0, together with those already available in mitoXplorer version 1.0, are shown in Figure 1 as well as Supplementary Figure S2.

### Novel functions in mitoXplorer 2.0

#### Mito-process enrichment analysis

In order to identify enrichment of our 38 manually curated mito-processes in a dataset, we implemented a gene set enrichment analysis (GSEA) function. This function helps guide users to focus on important mito-processes of a dataset for subsequent deeper analysis with other functions available in mitoXplorer 2.0.

Mito-process enrichment analysis was coded using the GSEApy Python library (7,8). When *P*-value and log_2_FC are both present, genes are ranked by the combined score as defined by Xiao *et al.* (9):

$$\begin{equation*}{\rm combined \, score} = {\rm abs}\left( {{\rm log}_2{\rm FC}} \right)\times - {\rm log}_{10}\left( {P \hbox{-} {\rm value}} \right).\end{equation*}$$

**Figure 2.**
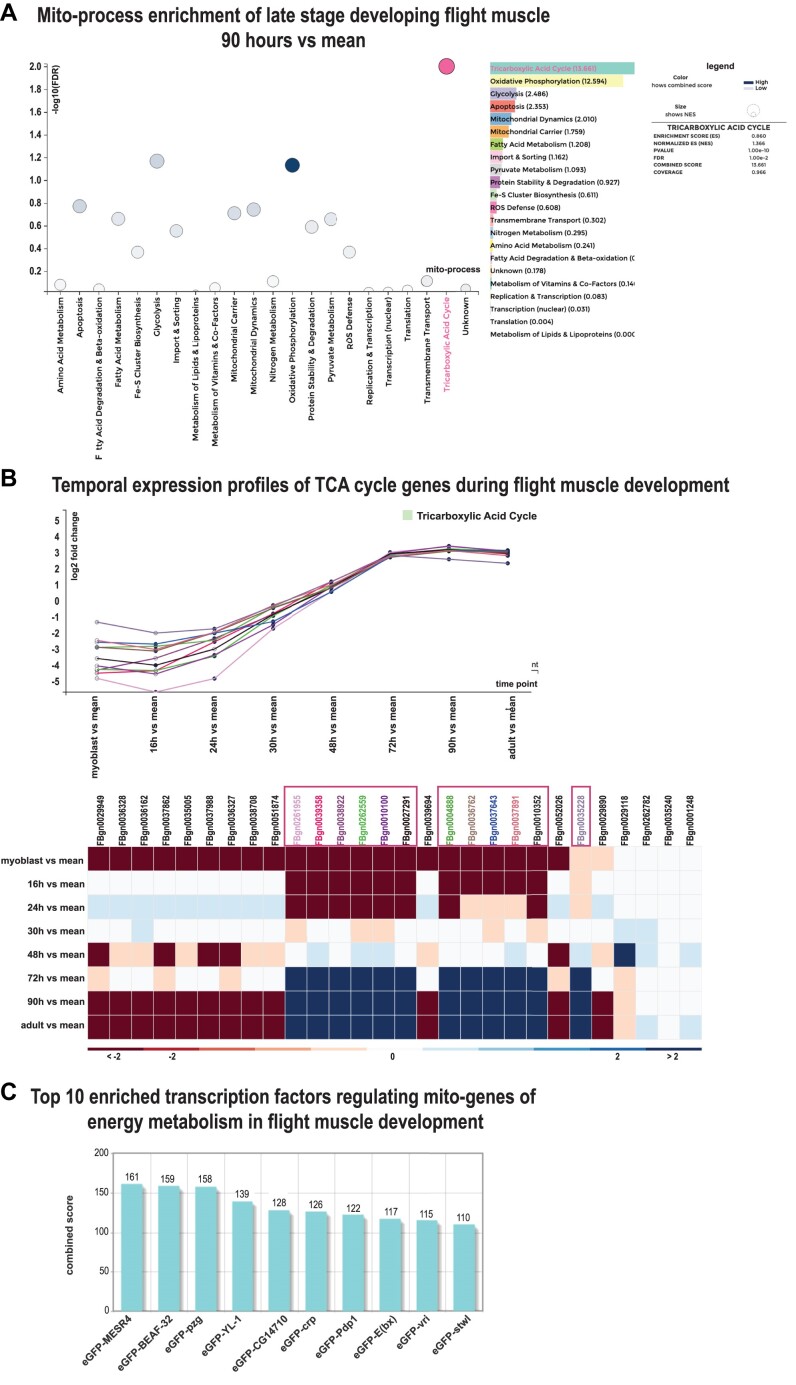
mitoXplorer 2.0 analysis of *D. melanogaster* developing indirect flight muscle (IFM). **(A)** Mito-process enrichment analysis of 90 h IFM after puparium formation (APF) revealed an enrichment of tricarboxylic acid (TCA) cycle, as well as OXPHOS. Mito-processes with a high combined score are highlighted in dark blue; the process TCA cycle has been selected and appears in pink. **(B)** Time-course visualization of developing *Drosophila* IFM, showing TCA cycle as an example. Genes that were downregulated early in development (myoblast, 30 h APF) and induced in late stages (72 h APF, adult) were selected for display in the line plot. Each visualized gene is shown in a different colour. **(C)** Top 10 enriched TFs potentially regulating mito-genes with the described expression profile (down early, up late) of the mito-processes OXPHOS, OXPHOS mt (mitochondrial-encoded genes) and TCA cycle. The plot is directly generated in AnnoMiner upon automatic upload of selected mito-genes within mitoXplorer 2.0’s time-course visualization function. See Supplementary Figure S3 as well as mitoXplorer’s video tutorials for instructions on how to generate these plots.

If no *P*-value is provided, genes are ranked by the absolute value of log_2_FC. The gene set enrichment score is tested against a null distribution of enrichment scores generated from 100 permuted gene sets composed of randomly selected genes from the input dataset. The results of the GSEA analysis are shown in the form of an interactive dot plot, as well as a bar plot (see Figure 2A for an example). In the dot plot, mito-processes are represented by bubbles, whereby the size reflects the normalized enrichment score and the colour represents the combined score. The *Y*-axis shows the significance of the test (−log_10_FDR), while the *X*-axis shows the different mito-processes. By clicking on one of the mito-process bubbles, all its values associated with the GSEApy analysis are displayed in the information panel. In the bar plot, mito-processes are ranked by combined score, whose value is also given in parentheses next to the mito-process.

#### Time-course visualization

In mitoXplorer 1.0, only six datasets could be compared to each other with the comparative plots. To overcome this limitation and to effectively visualize an increasing number of available time-course data, we implemented a time-course visualization interface. With this function, we allow users to visualize up to 20 time points of a temporal dataset. The structure of this visualization interface is similar to the comparative plots, with all its interactivity, except that genes can be individually selected from the sortable heatmap for display in the line plot by clicking on the gene name. Time points on the *X*-axis are connected via a line to visualize expression profiles over time. The user can thus visualize the temporal gene expression profile of a single or multiple mito-genes. Colours are assigned automatically to help visualize individual genes (see Figure 2B). This visualization was developed using the D3.js library (10) in order to produce a fully interactive visual interface.

#### Cross-species analysis

mitoXplorer 2.0 offers the possibility to compare datasets across species. Cross-species analysis is possible with the comparative plots or the heatmap function. This will help unravel similarities and understand differences in mitochondrial dynamics between species.

Orthology information has been included in the backend MySQL database of mitoXplorer by adding orthology ID tables for each model organism. These tables contain dictionaries, having as key the ID of the gene of the model organism of interest and as values the orthologous gene IDs in the other species available in mitoXplorer 2.0. Orthologs between the four model species offered by mitoXplorer were initially collected using the last release of BioMart (11). We manually curated these datasets and only 1:1 relations were kept.

The user has to first choose ‘cross-species’ from the ‘Data Mining’ drop-down menu and subsequently select the species to analyse. Only the comparative plot and the heatmap are offered for cross-species analysis. In the resulting plots of both visualization interfaces, the IDs of the first organism selected are used to label genes. However, the user can retrieve the ID of the ortholog of the second organism together with underlying information, by clicking on the respective cell in the heatmap or the bubble in the scatterplot.

#### Integrative analysis functions

In mitoXplorer 2.0, we also wanted to provide the possibility to identify mitochondrial regulators. More specifically, we wanted users to be able to find potential transcriptional regulators of co-regulated mito-genes, as well as identify potentially active signalling pathways from or to mitochondria. The interactivity of mitoXplorer allows one to directly select the gene(s) for both new integrative analysis functions starting from the comparative plot, heatmap or time-course visualization functions.

#### TF-enrichment

Identifying potential transcriptional regulators is done by performing a TF-enrichment analysis via connecting mitoXplorer to the AnnoMiner web server (5) and through direct upload of data from mitoXplorer to AnnoMiner. In brief, AnnoMiner seeks enriched peaks of transcriptional regulators from the ENCODE (12), modENCODE (13) and modERN (14) databases in the promoter regions of a user-provided gene set. This set of genes for an AnnoMiner *TF enrichment analysis* can directly be selected in mitoXplorer 2.0 and uploaded to the AnnoMiner web server via the TF-enrichment function (see Supplementary Figure S2E for the TF-enrichment menu). The selection can be done from three different interfaces: comparative plots, heatmap and time-course visualization. After invoking the ‘Integrative Analysis’ TF-enrichment, the user needs to select the genes by clicking on the boxes of the genes in the (sortable) heatmap (see Supplementary Figure S3 and our mitoXplorer video tutorials for detailed instructions). The user must then perform TF-enrichment using the AnnoMiner web server (a detailed user tutorial of AnnoMiner can be found at http://chimborazo.ibdm.univ-mrs.fr/AnnoMiner/tutorial.html and Supplementary Figure S3). As a result, the user receives a list of TFs that potentially regulate the selected mito-gene set within the AnnoMiner web server environment.

#### Network analysis

The integrative network analysis function aims at identifying active subnetworks (representing potential signalling paths) from and to mitochondria, starting from one selected mito-gene. To search for active subnetworks, mitoXplorer 2.0 first embeds the mito-interactome into the entire cellular interactome and then allows to integrate differential expression data with this cellular interactome. The user can then explore the expression dynamics of the network neighbourhood of a single selected mito-gene and, based on differential expression data, identify the active subnetwork surrounding this gene. This function can be invoked from the interfaces: comparative plots, heatmap and time-course visualization. To perform a network analysis, the user must first choose from the ‘Integrative Analysis’ panel the network analysis function (see Supplementary Figure S2F). Then, they can select a mito-gene to start network analysis by clicking on the box of the gene in the (sortable) heatmap, the maximum number of steps from the starting point (mito-gene), the log_2_FC threshold (to define a gene as deregulated or not) and whether unregulated nodes in the results are allowed (see Supplementary Figures S2 and S4 for the network analysis menu; see also our mitoXplorer tutorials for further instructions). Results will be displayed directly in a new window in the mitoXplorer platform.

The network analysis function has been developed using the NetworkX Python library (15). The cellular interactomes were created from the STRING database (v11) (16) using high-confidence physical protein–protein interactions and have been added to the MySQL database. The network neighbourhood of a selected gene is explored by using the ‘environment search’ algorithm described by the viPEr Cytoscape app (6). Once the analysis has been performed, the active subnetwork is drawn using the D3.js library (10).

#### IDconverter

In order to facilitate data upload, we added an IDconverter to allow usage of any gene identifier. This function converts input gene IDs (e.g. ENSEMBL, Entrez, RefSeq, Genecode, Flybase) on the fly to gene symbols, without any intervention from the user. The conversion is performed using the latest release of BioMart (11). BioMart data were downloaded and stored in text format. We retrieved them locally for reasons of speed.

#### Upgrade to Python

In order to enable local deployments and future development of the platform, we upgraded the backend of the web tool from Python 2.7 to Python 3.6.

The new mitoXplorer 2.0 menu is detailed in Supplementary Figure S2A–F; details on how to use new mitoXplorer 2.0 functions are shown in Supplementary Figures S3 and S4 and online at the mitoXplorer 2.0 tutorial pages (http://mitoxplorer2.ibdm.univ-mrs.fr/tutorials).

### Data processing for use cases 1 and 2

#### Flight muscle data (use case 1)

Temporal expression data of developing *D. melanogaster* IFM were taken from GSE107247 (17) in the form of raw read counts. In order to obtain differential expression values for the different time points versus the mean over all time points, we created two pseudo-replicates of the mean by first calculating the mean over all time points using one replicate per time point each. Normalization of the raw read counts as well as pairwise differential expression analysis was performed using DESeq2 (18).

#### Ataxia data (use case 2)

Data from the ataxia mouse model ATXN1_82Q_Tg from cerebellum were taken from GSE122099 (19). We downloaded sequencing reads and mapped them to mouse genome version mm10 using STAR (20) with default parameters. FeatureCounts (21) was used to calculate read counts. Normalization and differential expression analysis were done using DESeq2 via RNfuzzyApp (22). We compared all conditions to each other: 5-week wild type (WT) versus ATXN1_82Q_Tg, 12-week WT versus ATXN1_82Q_Tg, 5- versus 12-week WT, and finally 5- versus 12-week ATXN1_82Q_Tg. We uploaded the resulting data to mitoXplorer 2.0. For enrichment analysis of genes found in the active subnetworks extracted by mitoXplorer 2.0, we first downloaded and parsed the respective JSON files to obtain gene lists. These were then submitted to EnrichR (7); for further downstream analysis, we used the KEGG resource (23).

## RESULTS

### Use case 1: identifying transcriptional regulators of mito-genes during *D. melanogaster* indirect flight muscle development

We analysed temporal mito-gene expression dynamics during *D. melanogaster* IFM development using the new mitoXplorer 2.0 functions. In the developing indirect flight muscle, mitochondria undergo significant structural, but also bioenergetic changes (17,24). We wanted to explore these mitochondrial changes further using mitoXplorer 2.0. We used a transcriptomic resource generated by Spletter *et al.*, consisting of transcriptomic data from *Drosophila* isolated IFM at myoblast stage, 16, 24, 30, 48, 72 and 90 h after puparium formation (APF), and the adult stage (17). To observe temporal changes during IFM development, we compared each time point to the mean over all time points (see the ‘Materials and Methods’ section) and uploaded the data to mitoXplorer 2.0.

We first investigated which mito-processes are enriched during IFM development and used the new mito-process enrichment function. We used nearly mature IFM at 90 h APF for mito-process enrichment analysis. We found mito-processes related to OXPHOS-dependent energy metabolism enriched at this time point (Figure 2A and Supplementary Figure S3A).

We next investigated the temporal expression profiles of mito-genes from the mito-processes OXPHOS, OXPHOS mt and TCA cycle using the time-course visualization function of mitoXplorer 2.0. As exemplified in Figure 2B, we found several mito-genes within these three mito-processes that showed a specific temporal expression profile, being first downregulated (myoblast stage, 16, 24 and 30 h APF) and subsequently strongly induced from 72 h APF to the adult stage (see also Supplementary Figure S3B).

We were interested in identifying potential TFs responsible for regulating these co-regulated genes. We therefore made use of mitoXplorer 2.0’s new integrative analysis function TF-enrichment. We selected all genes from the three mito-processes that showed the above-described temporal expression profile and uploaded them via mitoXplorer’s TF-enrichment function to AnnoMiner (see Supplementary Table S1A for the list of genes selected and Supplementary Figure S3C and D). AnnoMiner returned several interesting TFs as enriched in the promoters of the co-regulated mito-genes (Supplementary Table S1B). Among the top 10 hits are *MESR4*, which has been shown to be involved in development and in the cellular response to hypoxia (25); *pzg*, which is known to regulate developmental processes (26,27); *Pdp1*, which is known to regulate muscle genes (28); crp, which controls cell growth and tracheal terminal branching (29); or *vri*, which has been shown to be involved in tracheal development (30,31).

To conclude, this use case demonstrates how the new functions of mitoXplorer 2.0, mito-process enrichment, time-course visualization and the integrative analysis function TF-enrichment could help identify the gene expression dynamics behind the mitochondrial bioenergetic switch in developing *Drosophila* indirect flight muscle and predict potential transcriptional regulators responsible for this switch.

### Use case 2: identification of signalling cascades regulating Ca^2+^ signalling in a mouse model of spinocerebellar ataxia type 1

Spinocerebellar ataxias (SCAs) are a group of dominantly inherited neurodegenerative diseases, which are defined by a loss of coordination of body movements and cerebellar degeneration. They can be caused by mutations in nearly 40 genes and are currently untreatable. A disruption of Ca^2+^ signalling in cerebellar cells, and more specifically in the Purkinje neurons, is considered to play a key role in disease onset and progression (32). We wanted to explore the contribution of mitochondria to Ca^2+^ signalling deregulation in SCAs. To this end, we used a mouse model of spinocerebellar ataxia type 1 (SCA1). SCA1 affects the cerebellum as well as the inferior olive and is caused by polyglutamine expansion in the *ATAXIN1* gene, which encodes a transcriptional regulator (33). We used RNA expression data from a mouse model of SCA1 from (19), which looked at cerebellum and inferior olive of 5- and 12-week-old transgenic ATXN1_82Q (ATXN1_82Q_Tg) mice. We focused our analysis on the cerebellum and were mostly interested in the temporal differences of pathways possibly affecting mitochondrial Ca^2+^ signalling and transport.

**Figure 3.**
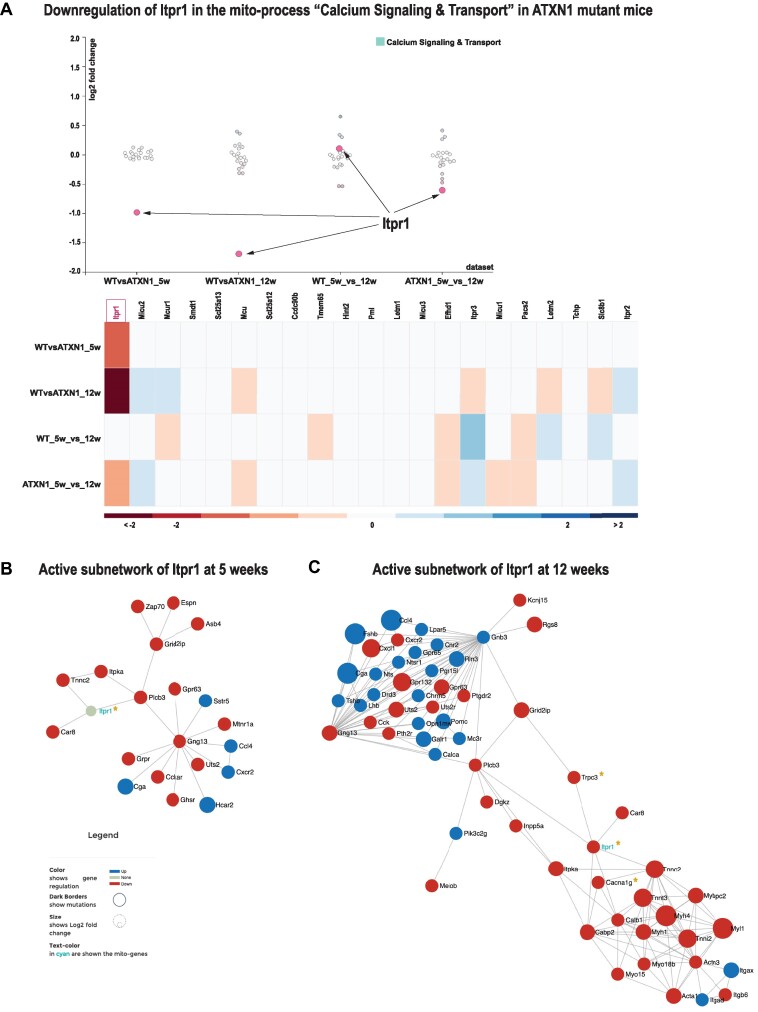
Ca^2+^ signalling and transport is affected in SCA1. **(A)** One of the most affected genes in the ATXN1_Q82 transgene mouse model for SCA1 is *Itpr1*, which localizes to the ER and regulates mitochondrial Ca^2+^ signalling. **(B)** Network neighbourhood of *Itpr1* in 5-week-old ATXN1_Q82_Tg mice. **(C)** Network neighbourhood of *Itpr1* at 12 weeks. Genes found to cause a form of SCA are marked with an asterisk. Mito-genes are labelled in cyan, while non-mitochondrial genes are labelled in black. The user can interactively change gene positions and labels in order to show different information (gene name or ID, mutation, log_2_FC). The visualization is information dense. Node size, colour and border represent magnitude of log_2_FC, direction of the deregulation (up or down) and mutation information, when available. See Supplementary Figure S4 as well as mitoXplorer’s video tutorials for instructions on how to generate these plots.

We uploaded differential expression data of ATXN1_82Q_Tg compared against WT at 5 and 12 weeks, as well as the temporal comparisons of WT (5 and 12 weeks) and ATXN1_82Q_Tg (5 and 12 weeks), to mitoXplorer 2.0. We used comparative plots to visualize differences in gene expression in ‘Ca^2+^ signalling and transport’ in those four datasets. Among the genes most affected in this mito-process was *Itpr1* (Figure 3A and Supplementary Figure S4A), an intracellular receptor for inositol 1,4,5-trisphosphate that is located at the endoplasmic reticulum (ER). *Itpr1* is one of the main regulators of mitochondrial Ca^2+^ signalling (34) and is itself regulated by mitochondria (35). Furthermore, mutations in *ITPR1* cause SCA15 (36).

We next wanted to know how the network neighbourhood and thus potential signalling pathways change between the two disease stages. We therefore made use of the new integrative network analysis function in mitoXplorer 2.0 (see Supplementary Figure S4B). We extracted active subnetworks surrounding the *Itpr1* gene using differential expression data of 5- and 12-week-old ATXN1 mice, respectively, and then compared them first by visual inspection (Figure 3B and C; no deregulated nodes allowed, log_2_FC ≥ |1.2|, step size 3). The extracted active subnetwork surrounding *Itpr1* is substantially larger at 12 weeks with more differentially expressed nodes within a three-step neighbourhood (Figure 3C) compared to the earlier stage (Figure 3B). We then downloaded the two extracted, active subnetworks (Supplementary Figure S4C) for enrichment analysis using EnrichR and KEGG. Many of the enriched pathways overlap between the two time points, such as calcium signalling, neuroactive ligand–receptor interaction or phosphatidylinositol signalling. At 12 weeks, some more metabolic pathways as well as SCA appear to be enriched (Supplementary Table 2B–D). Moreover, three genes responsible for SCAs are found in the 12-week network, including *Itpr1* itself, together with *Trpc3* and *Cacna1g* (Supplementary Table S2E).

In conclusion, we used the comparative plots and network analysis functions to investigate Ca^2+^ signalling deregulation in an SCA1 mouse model and found several signalling pathways consistently induced from 5 to 12 weeks in the cerebellum. Moreover, two more genes related to SCA were found in the 12-week active subnetwork.

## DISCUSSION

Here, we present an important upgrade of the mitoXplorer visual data mining platform. While mitoXplorer version 1.0 (1) allowed to inspect mitochondrial datasets in multiple ways, mitoXplorer 2.0’s integrative analysis functions permit the user to go beyond simple data visualization. Our new integrative functions can be used to better understand the interaction between mitochondria and their cellular environment, as we have shown in two of our use cases. In the first use case, we showed how the new functions of mitoXplorer 2.0 (the mito-process enrichment, time-course visualization and TF-enrichment) can be used to identify key transcriptional regulators of important mitochondrial processes. In the second one, we employed network analysis, which, by integrating the mitochondrial interactome into the complete cellular interactome, allows the identification of important non-mitochondrial genes that are connected with and potentially regulate mitochondrial processes. These results demonstrate the power of mitoXplorer 2.0’s downstream and integrative analysis functions.

For TF-enrichment, we decided to rely on AnnoMiner (5). By connecting mitoXplorer 2.0 to AnnoMiner, direct data transfer from one to the other allows us to execute AnnoMiner’s *TF enrichment analysis*, to identify potential transcriptional regulators of mitochondrial functions. To date, several tools for TF-enrichment are available, but to our knowledge only few of these, namely modEnrichr (37), iCisTarget (38) and AnnoMiner, work on model organisms other than human and mouse and thus can be used for three out of the four model organisms present in mitoXplorer 2.0: human, mouse and *D. melanogaster*. AnnoMiner has also data for *Caenorhabditis elegans*, a model species we plan to offer soon on mitoXplorer 2.0. For network analysis, we implemented a function for active subnetwork extraction starting from a single gene based on the algorithm of the Cytoscape app viPEr (6). The exploration of the network neighbourhood of a gene using viPEr requires only minimal user input: a selected gene; the number of steps from this gene for extracting an active subnetwork; a log_2_FC threshold for defining genes as deregulated; and finally, whether to accept connecting genes that are not deregulated. The algorithm thus avoids cumbersome tuning of parameters. The fact that we implemented this algorithm using NetworkX (15) and D3.js (10) permits a fully interactive exploration of the extracted subnetwork, facilitating its interpretation. In the future, we plan to include downstream analysis of the resulting network, such as pathway annotation through enrichment analysis. This should help to better identify potential signalling paths regulating mitochondrial functions. We find these two integrative analysis functions as an important novel upgrade of mitoXplorer, which should help to unravel how mito-gene expression and thus mitochondrial functions adjust to a specific cellular environment.

Additional future developments we are currently working on include mitochondrial metabolic modelling using flux balance analysis as well as logical modelling. Combining mitoXplorer directly with metabolic modelling would allow users to assess the metabolic states of biological samples based on mito-gene expression data directly within the mitoXplorer platform. Starting mitochondrial models are available for human and budding yeast (39–41), which we will adapt to our needs. In addition, we plan to extend mitoXplorer to allow analysis of single-cell RNA-seq data of tissues on cell-type level. All these further implementations are simplified by the modular fashion in which mitoXplorer 2.0 has been developed.

## DATA AVAILABILITY

mitoXplorer 2.0 is available as a web server at http://mitoxplorer2.ibdm.univ-mrs.fr. The source code together with installation instructions is available in our GitLab repository (https://gitlab.com/habermann_lab/MitoX2).

Public gene expression data used for this study were obtained from the Gene Expression Omnibus repository at NCBI (42). *Drosophila* developmental IFM time-course data have been downloaded from GSE107247 (17). Mouse ATXN1 raw sequencing data have been downloaded from GSE122099 (19).

## Supplementary Material

gkac306_Supplemental_Files
