# Supplementary material for: The mitoXplorer 2.0 update: integrating and interpreting mitochondrial expression dynamics within a cellular context
Source: Nucleic Acids Res. 2022 May 7;50(W1):W490–9. doi: 10.1093/nar/gkac306 (PMC9252804; doi:10.1093/nar/gkac306)
Supplement: gkac306_Supplemental_Files [file gkac306_supplemental_files.zip › Marchiano_etal_SupplementaryData_R1.pdf]

The mitoXplorer 2.0 update: integrating and interpreting mitochondrial expression dynamics within a cellular context

Marchiano Fabio[1], Haering Margaux[1], Habermann Bianca Hermine[1*]

[1] Aix-Marseille University, CNRS, IBDM UMR 7288, 13009 Marseille, France
[*] to whom correspondence should be addressed (bianca.habermann@univ-amu.fr)

Supplementary Data:

Use case 3 and Supplementary Figure S1: Comparison of differential expression in ROS defense from fibroblasts of human Trisomy 21 with fibroblasts from a mouse model for Trisomy 21.

Supplementary Figure S2: New mitoXplorer 2.0 menu. Detailed description of the new menu of mitoXplorer 2.0.

Supplementary Figure S3: Steps to perform Mito-Process Enrichment, Time-course Visualisation and Transcription Factor Enrichment as demonstrated in Figure 2 of the main manuscript.

Supplementary Figure S4: Steps to use Comparative Plots and perform Network Analysis as demonstrated in Figure 3 of the main manuscript.

Supplementary Table S1: (a) *Drosophila* gene lists uploaded to AnnoMiner. **(b)** Enriched TFs identified for **(a)** by AnnoMiner.

Supplementary Table S2: (a) Gene lists of 5- and 12-weeks active subnetworks of the ATXN1_82Q Tg mouse model. **(b)** Enriched KEGG pathways of 5 weeks ATXN1_82Q Tg gene list. **(c)** Enriched KEGG pathways of 12 weeks ATXN1_82Q Tg gene list. **(d)** KEGG pathways with >3 genes from the 5 week and 12-week networks. **(e)** Genes related to Spinocerebellar Ataxias in the 5 week and 12-week network**.**

Use case 3: Comparison of differential expression in Protein Stability & Degradation from fibroblasts of human Trisomy 21 with fibroblasts from a mouse model for Trisomy 21

We wanted to know, whether differential expression patterns of mito-genes are similar in fibroblasts from human monozygotic twins discordant for Trisomy 21 model and a mouse model of Trisomy 21. We used data from (1) , which were already uploaded to mitoXplorer and invoked the Cross-species function of mitoXplorer 2.0. We used the comparative Plots to compare the two datasets of trisomic fibroblasts, one from human monozygotic twins discordant for Trisomy 21, and the other from fibroblasts of a mouse model of Trisomy 21, Ts65DN. Overall, we found good overlap between the two species in most of the genes, with a few exceptions, where we saw differential expression in opposite directions. We show as an example the process Protein Stability & Degradation, in which all orthologous genes show the same differential expression behavior in the human and mouse Trisomy 21 model. It was already observed by (2) that protein turnover is enhanced in Trisomy 21 cells. The fact that several factors involved in protein stability and turnover are de-regulated fits well with this observation and seems to be conserved between human and mouse.

Supplementary Figure S1

Cross-species comparison of human Trisomy 21 fibroblasts against fibroblast from a Trisomy 21 mouse model (Ts65DN)

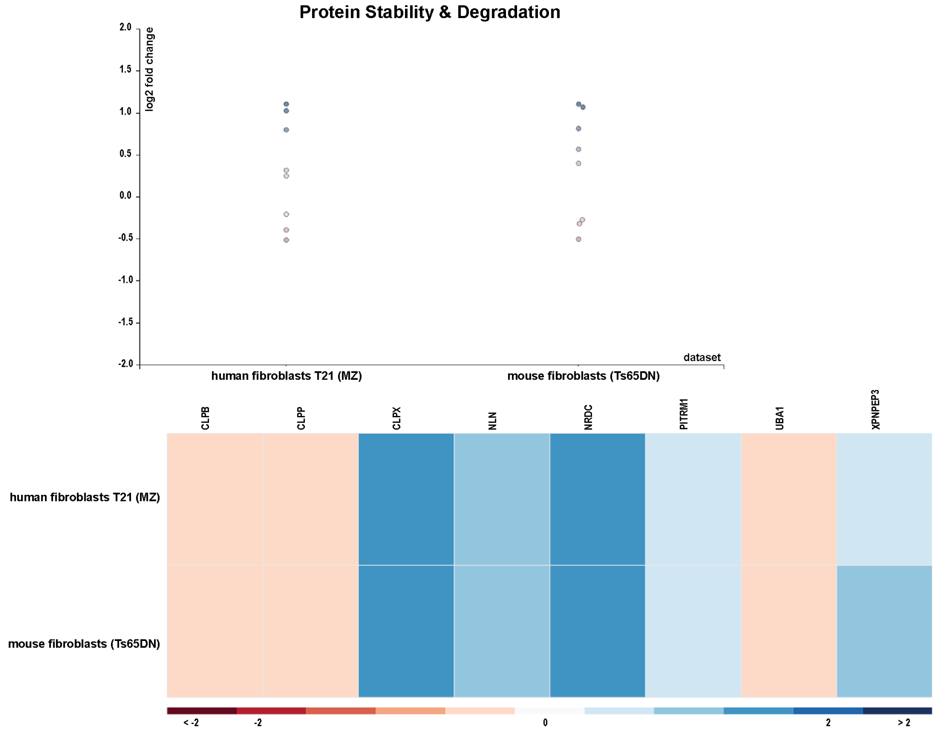

Figure S1: Cross-species comparison of differential expression data between data from human fibroblasts from monozygotic twins discordant for Trisomy 21 and fibroblasts from a mouse model for Trisomy 21 (Ts65DN). Differential expression data from human and mouse Trisomy 21 fibroblasts were compared against their respective wild-type controls (data available in mitoXplorer (3)). Red color indicates downregulation, blue color up-regulation. The gene NGB has been selected in the scatterplot.

Supplementary Figure S2

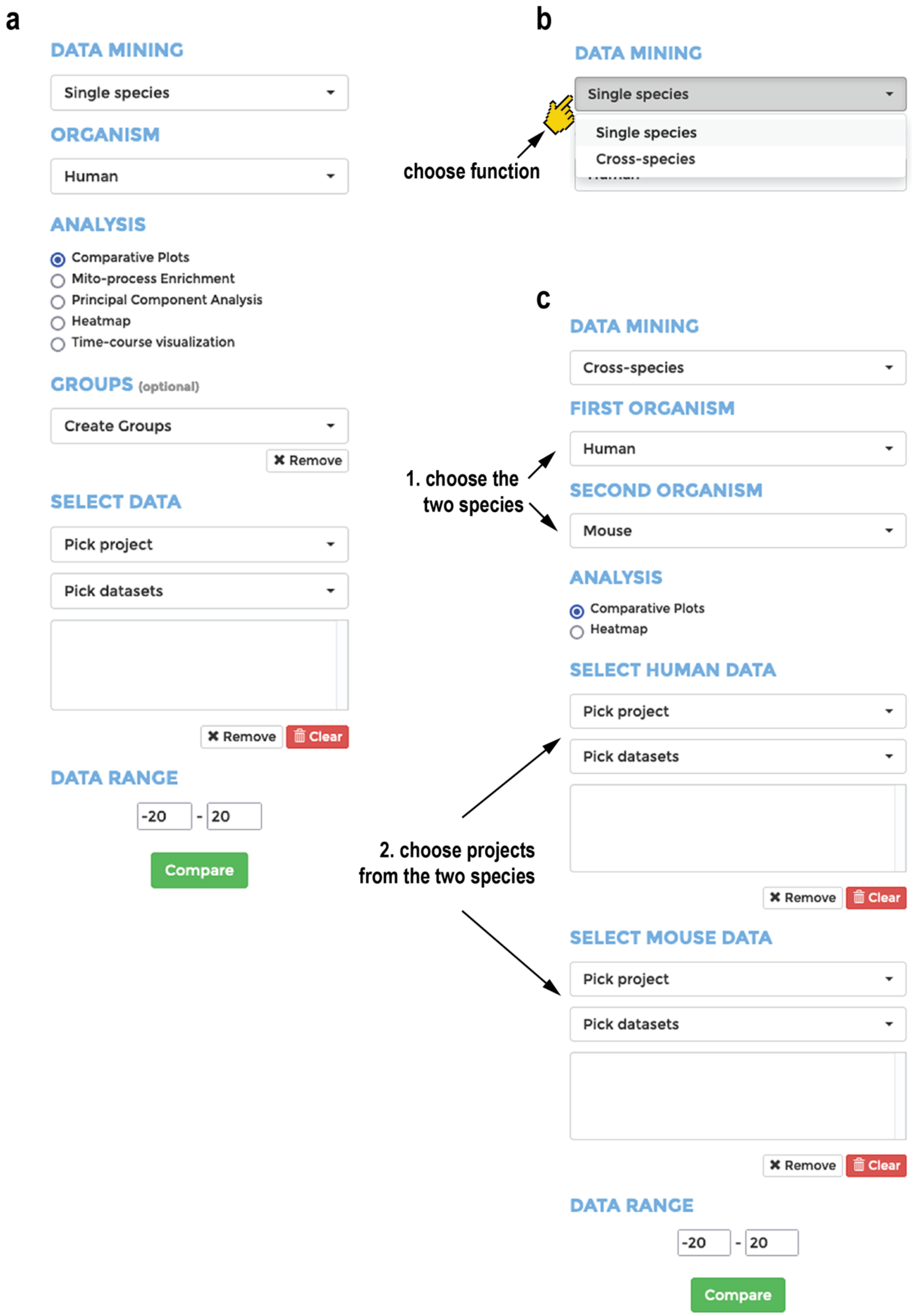

Supplementary Figure S2

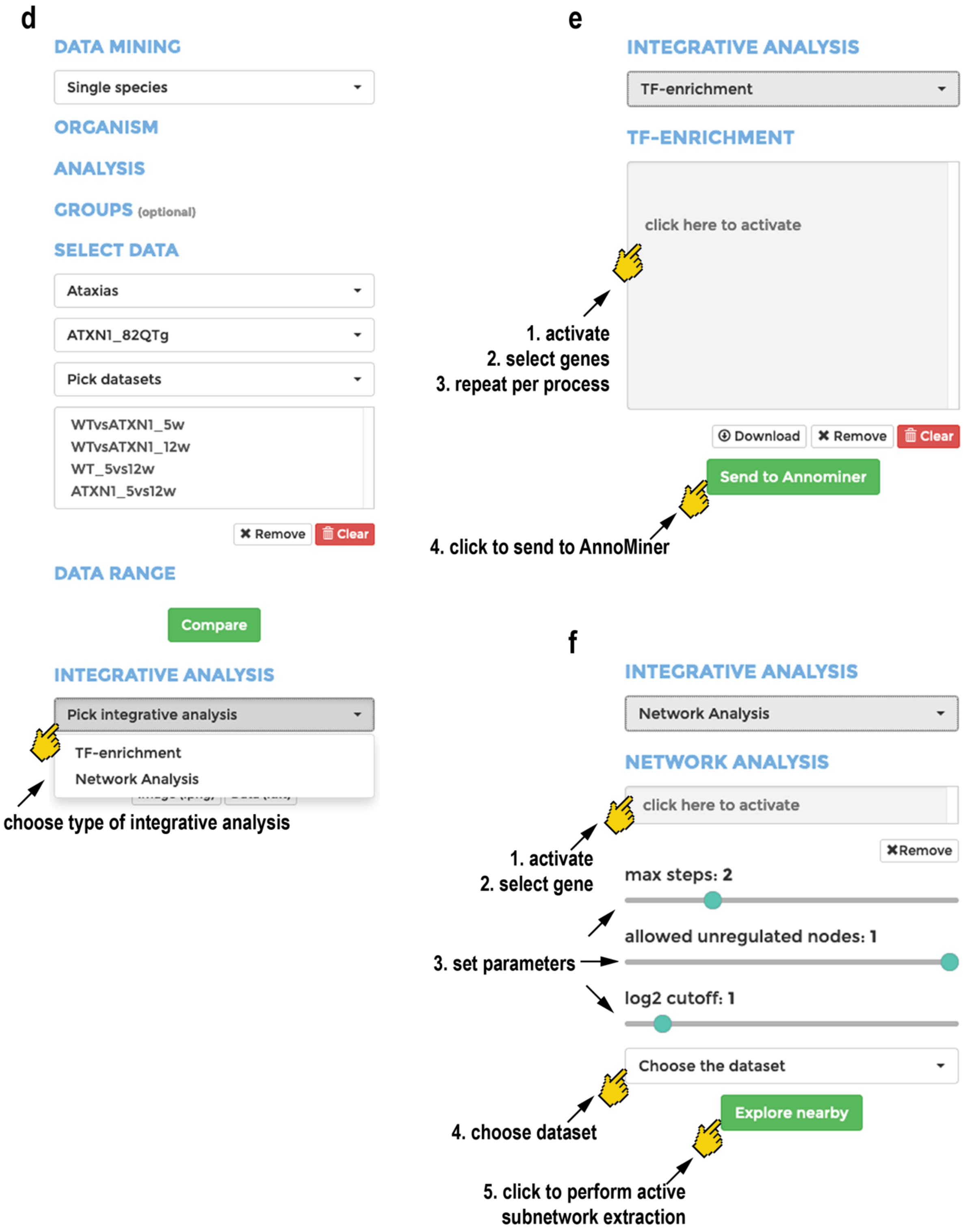

Supplementary Figure S2: New menu of mitoXplorer 2.0. (a) New menu functions of mitoXplorer main menu includes the DATA MINING panel for choosing between single species and cross-species; New functions in the ANALYSIS panel include Mito-process Enrichment and Time-course Visualisation. **(b)** When clicking on the drop-down menu in DATA MINING, the user can choose between Single species and Cross-species. **(c)** When choosing Cross-species analysis, 1. the two organisms have to be chosen; and 2. the datasets to be compared from both organisms have to be chosen. As analysis functions, Comparative Plots as well as Heatmaps are offered **(d)** Once a dataset has been uploaded, the INTEGRATIVE ANALYSIS panel appears. From here, the user can choose in a drop-down menu TF-enrichment and Network Analysis **(e)** When choosing TF-enrichment, a window appears with

the text **‘click here to activate**’ that needs to be clicked (1.), before genes can be selected (2.). When genes from a second mito-process should be added, the window **needs to be clicked again** (3.). Once the user is satisfied with the selected gene list, it can be sent to AnnoMiner by clicking on the green box called ‘Send to AnnoMiner’ (4.). The gene list can also be downloaded when clicking on the box called ‘Download’. The user will be directed to AnnoMiner to look for enriched TFs (for details on the usage of AnnoMiner, see (4) and also the video instructions provided on the AnnoMiner web-site (http://chimborazo.ibdm.univ-mrs.fr/AnnoMiner/tutorial.html). **(f)** When Network Analysis is chosen as downstream analysis, several items appear: a box containing the text **‘click here to activate**’ that must be clicked to be activated before (1.) a single gene can be selected (2.); a set of parameters (3.), which include the maximal step size of the resulting active subnetwork, the number of nodes in the paths allowed without being differentially expressed; and the log2FC cutoff that defines differentially expressed genes; a drop-down menu from which a dataset to be used for the active subnetwork extraction has to be chosen (4.); finally, by clicking on the green box called ‘Explore nearby’, the gene together with the dataset and chosen parameters will be sent to the subnetwork extraction function. A resulting network will appear in a separate browser window within the mitoXplorer platform.

Supplementary Figure S3

Steps to perform Mito-Process Enrichment Analysis, Time-Course Visualisation and Transcription Factor Enrichment as shown in Figure 2.

a. How to perform Mito-process Enrichment

a.1. menu selections

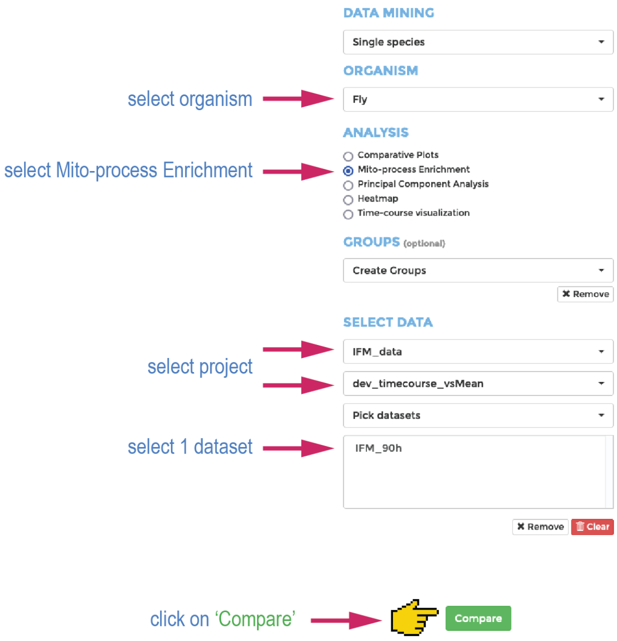

a.2. interactive results

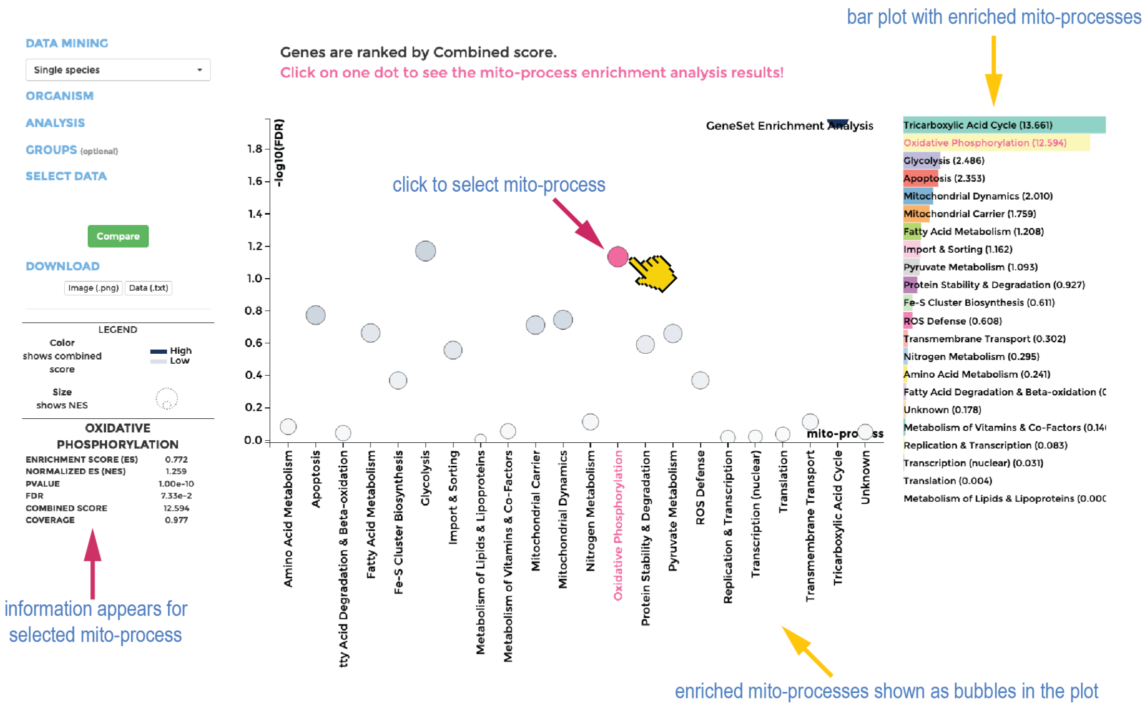

b. How to use Time-course Visualisation

b.1. menu selections

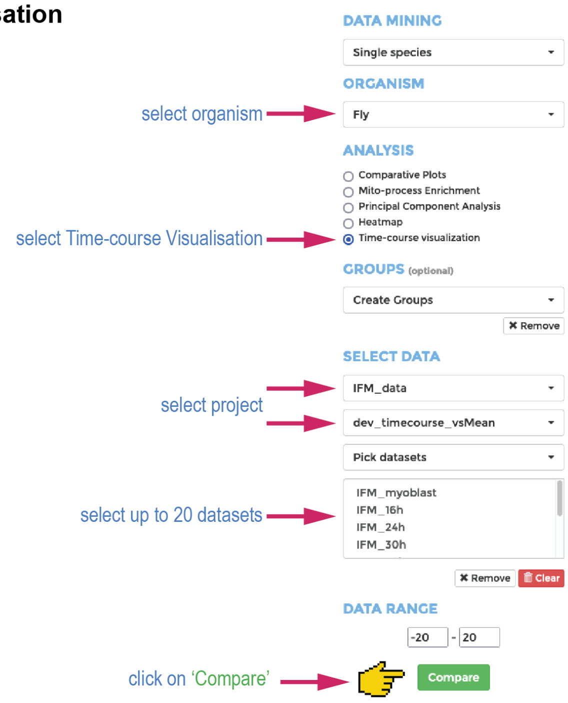

b.2. interactive results

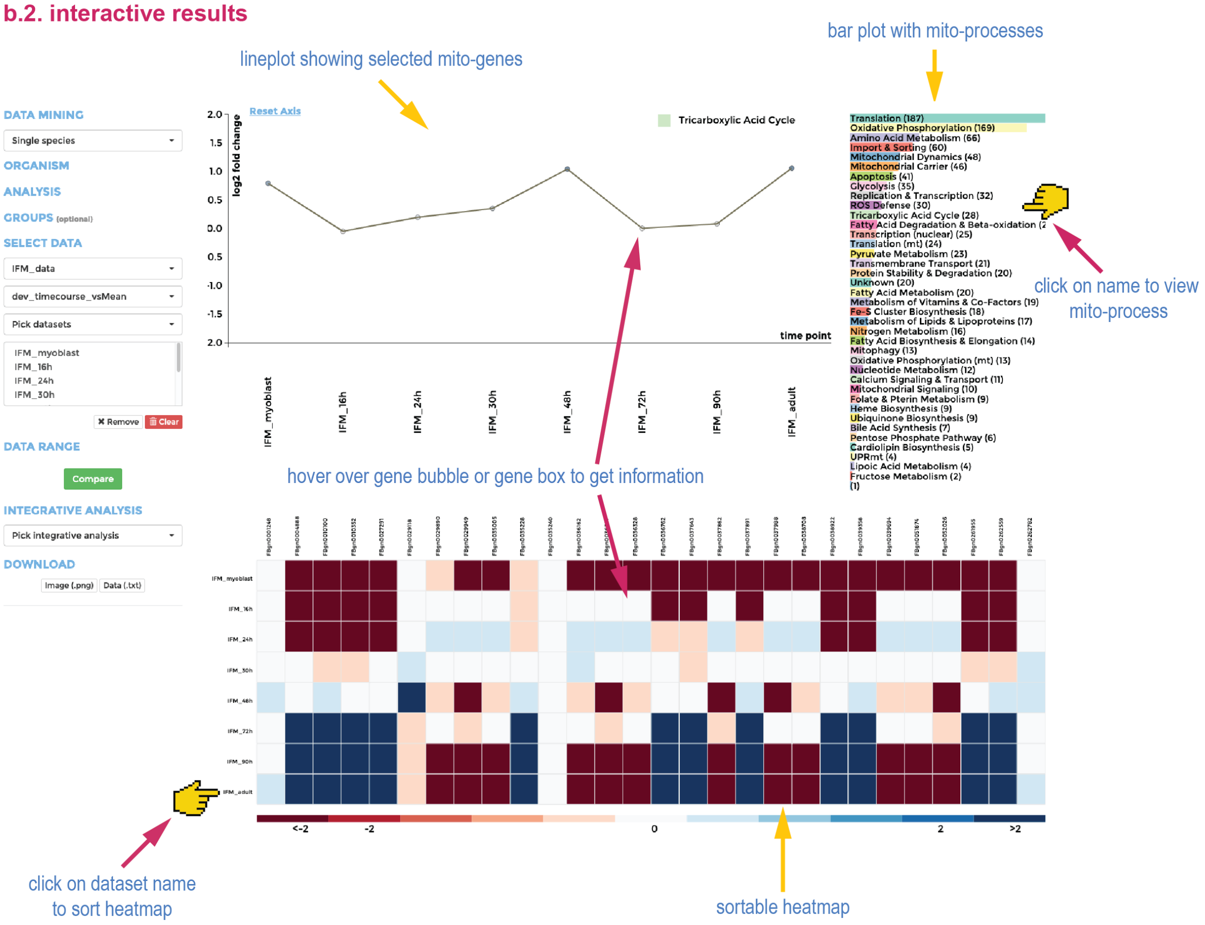

b. How to use Time-course Visualisation

b.3. select genes for display in lineplot

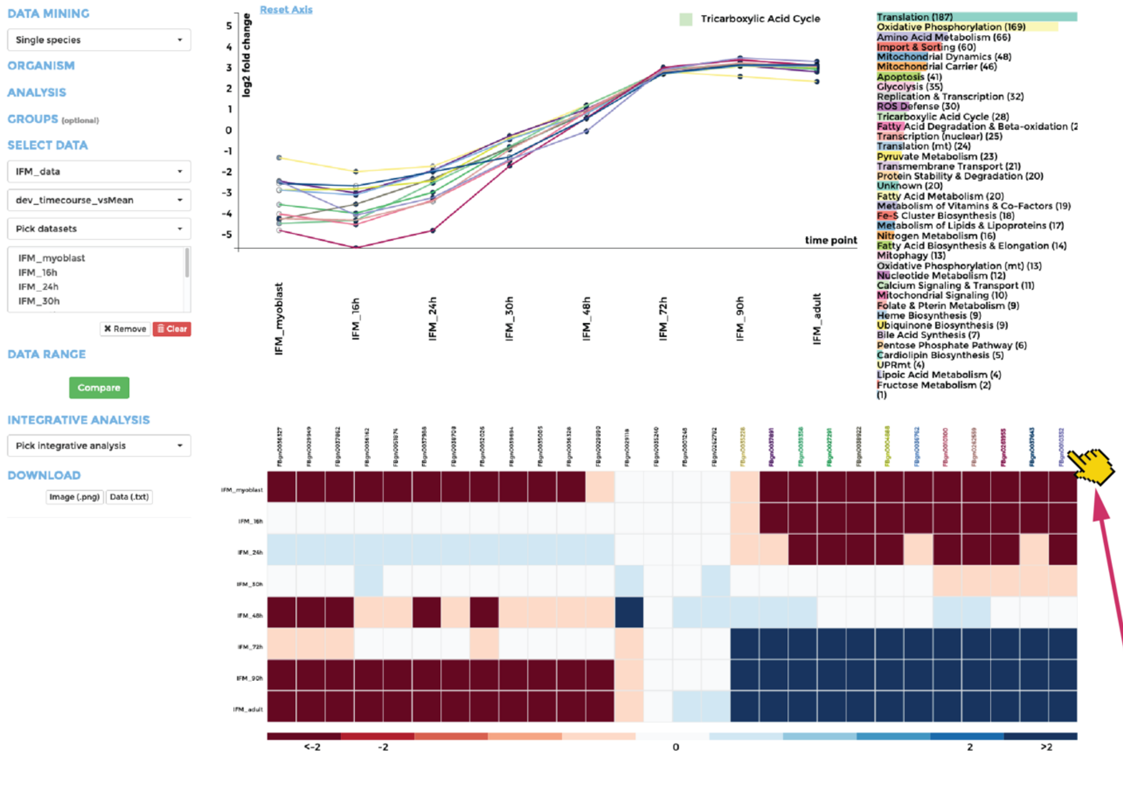

click on gene names to select or deselect genes to be shown in the lineplot

c. How to select genes for TF-enrichment

c.1. menu selections

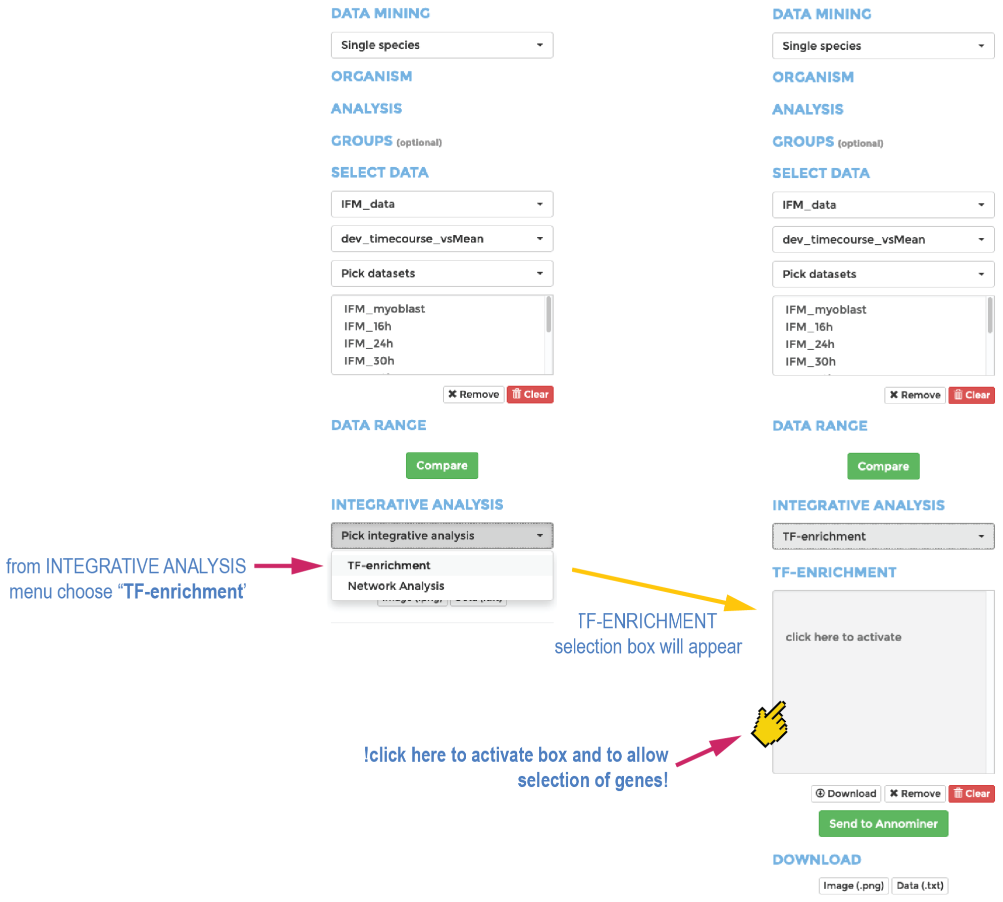

c.2. select genes from sortable heatmap for TF-enrichment

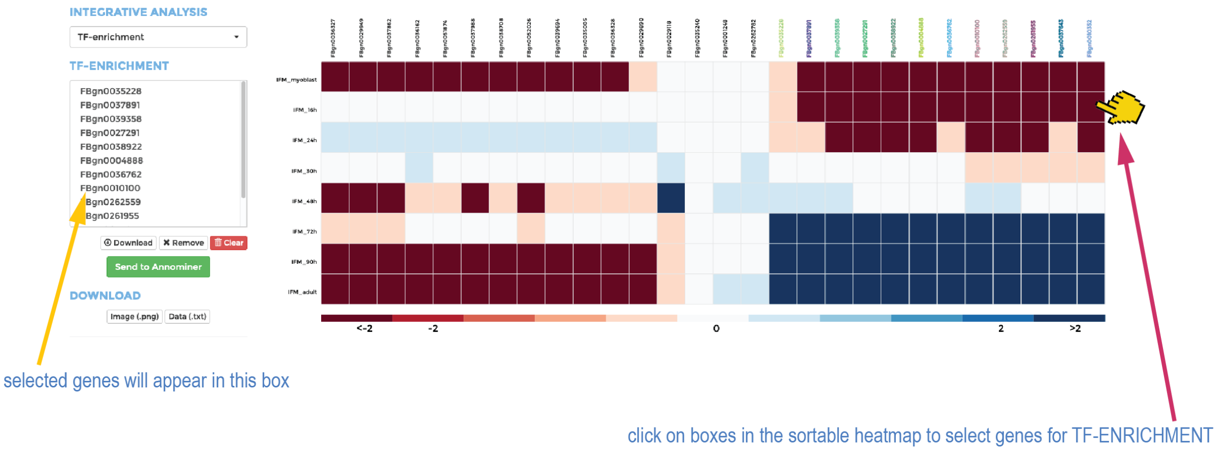

c. How to select genes for TF-enrichment

c.3. add genes from additional processes and submit to AnnoMiner

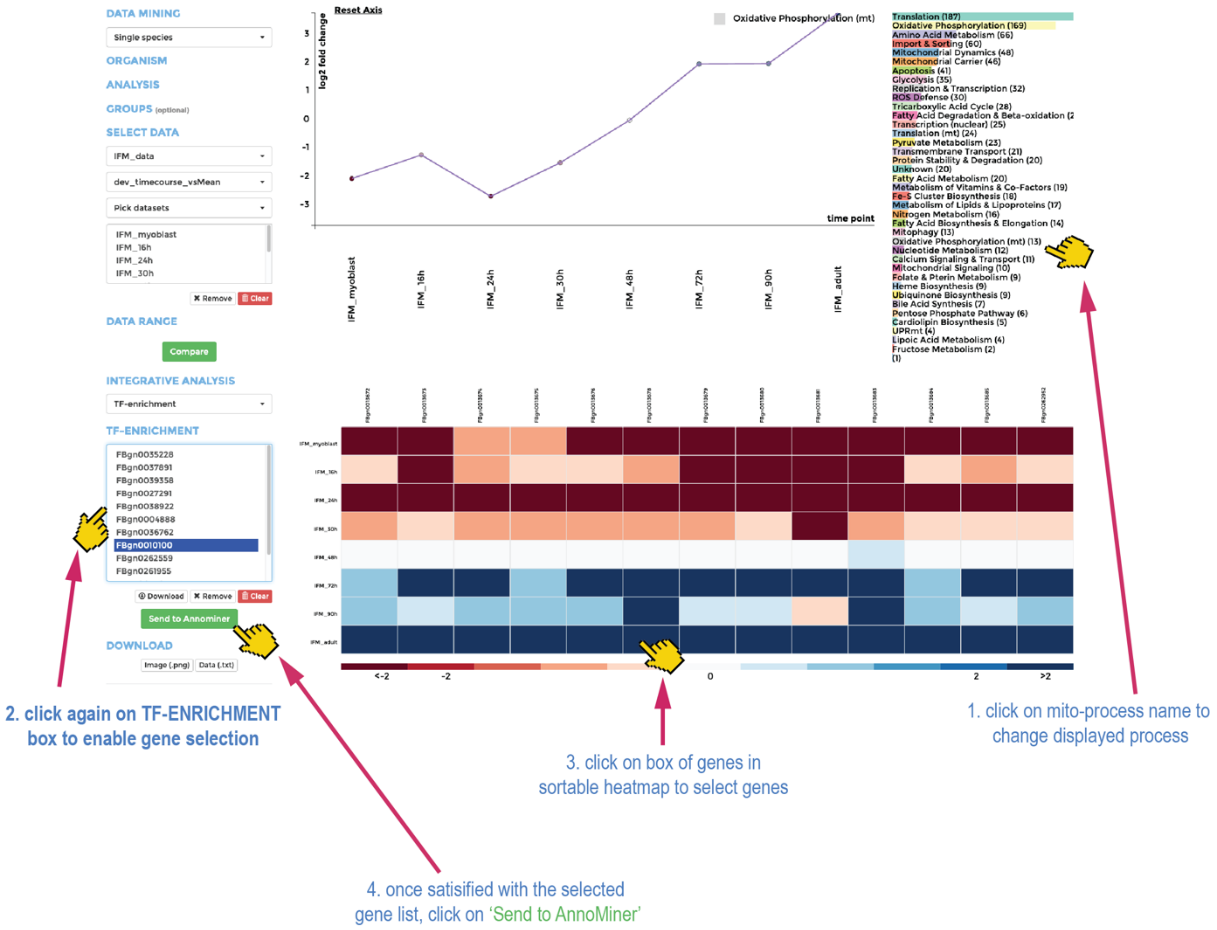

d. How to perform TF-Enrichment Analysis in AnnoMiner

d.1. select parameters for AnnoMiner TF-enrichment analysis

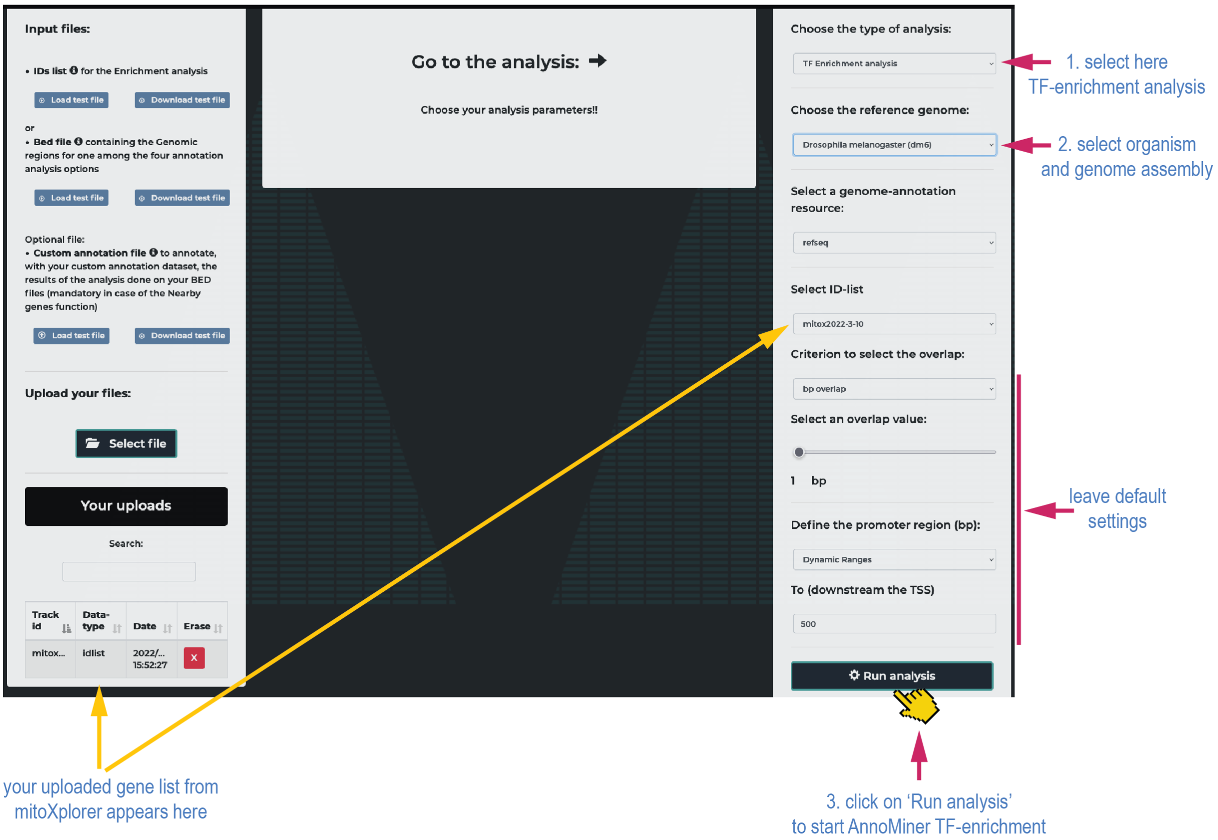

d. How to perform TF-Enrichment Analysis in AnnoMiner

d.2. AnnoMiner TF-enrichment analysis results

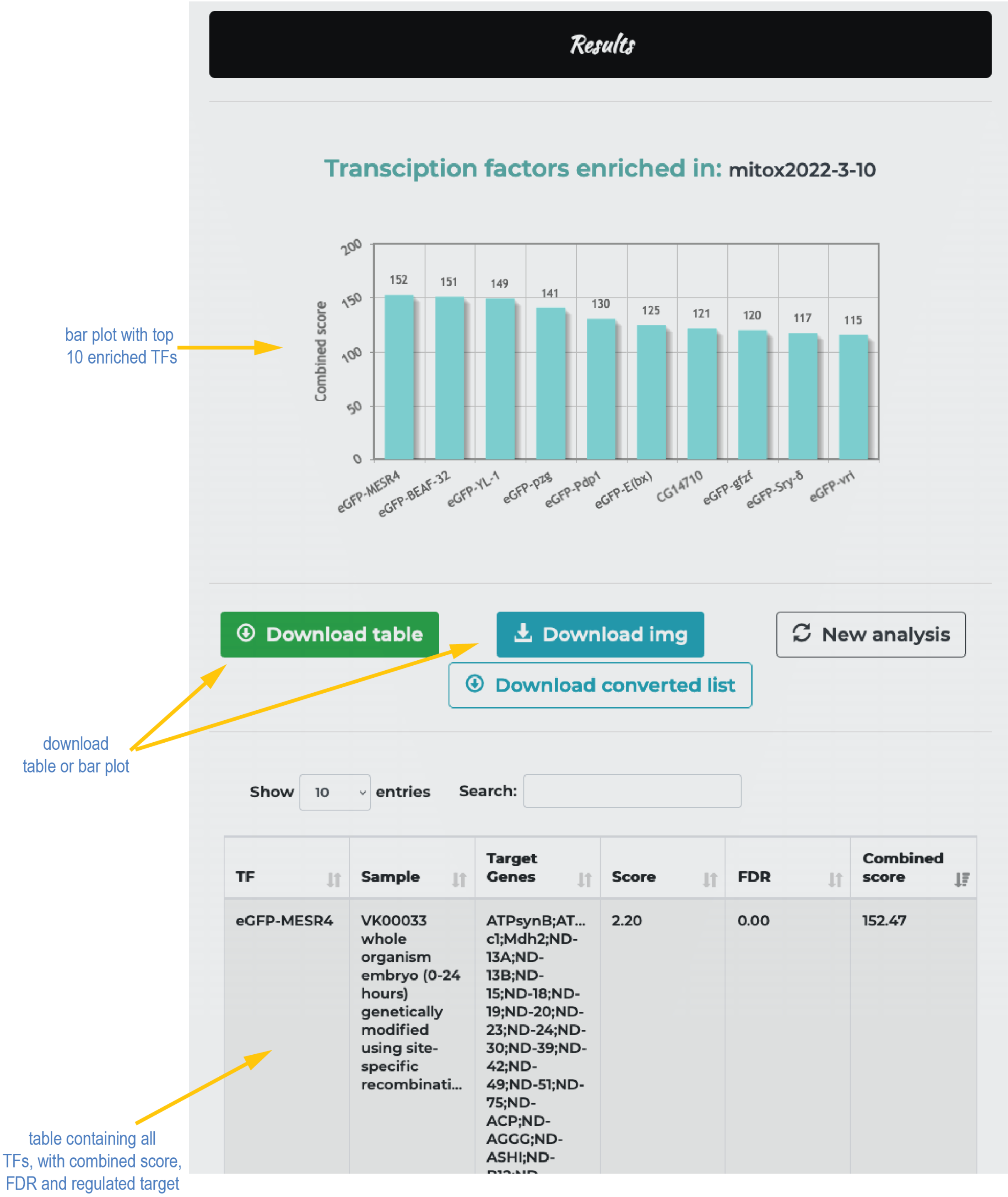

bar plot with top 10 enriched TFs

download table or bar plot

table containing all TFs, with combined score, FDR and regulated target genes

Supplementary Figure S4

Steps to use Comparative Plots and perform Network Analysis as shown in Figure 3

a. How to use Comparative Plots

a.1. menu selections

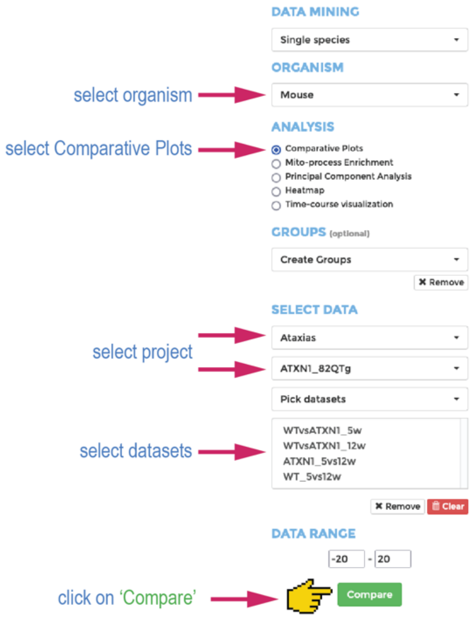

a.2. interactive results and selecting network analysis

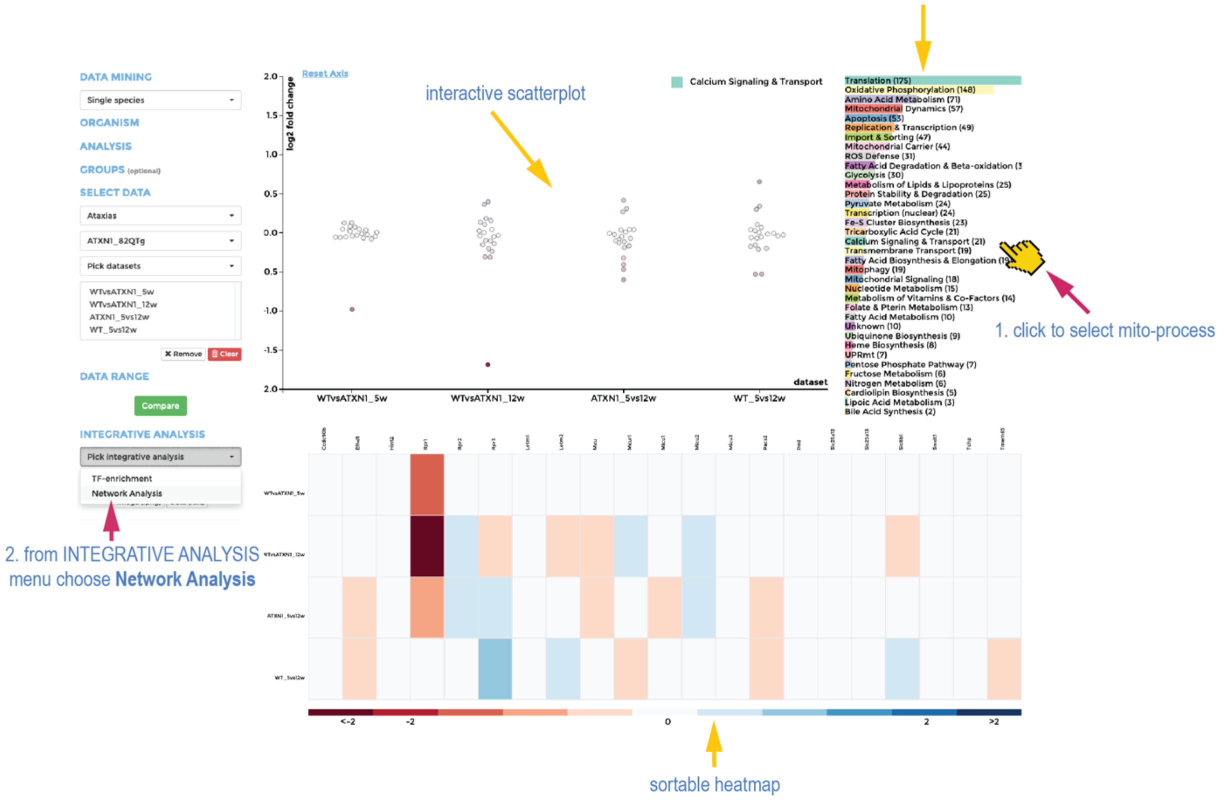

b. How to select genes for Network Analysis

b.1. selection menu for Network Analysis

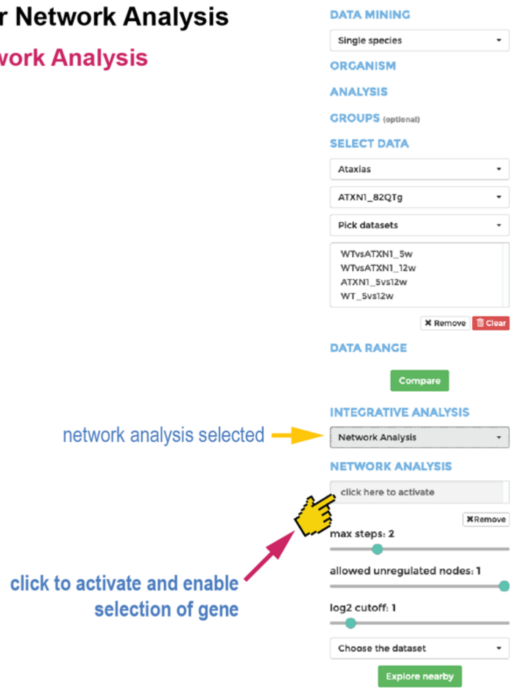

b.2. interactive results and initiating network analysis

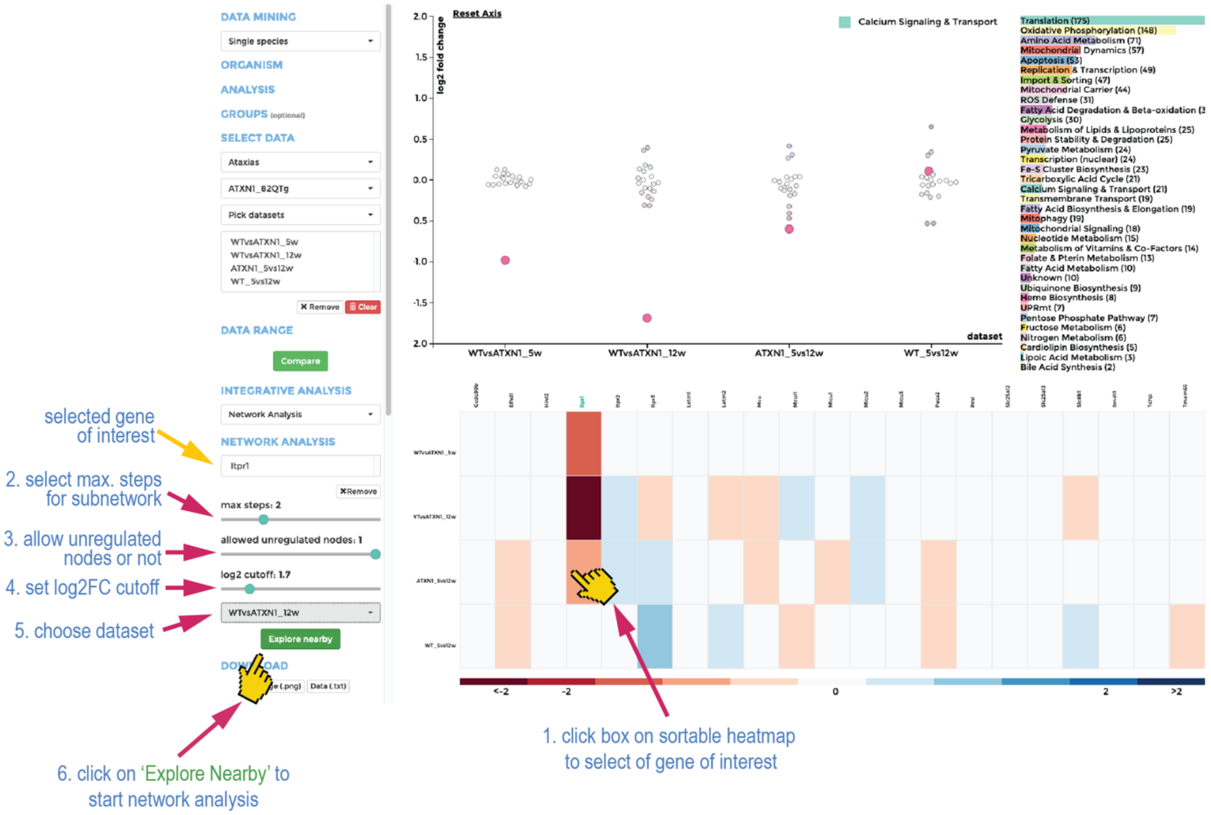

c. Network Analysis visualisation

c.1. interactive network analysis results

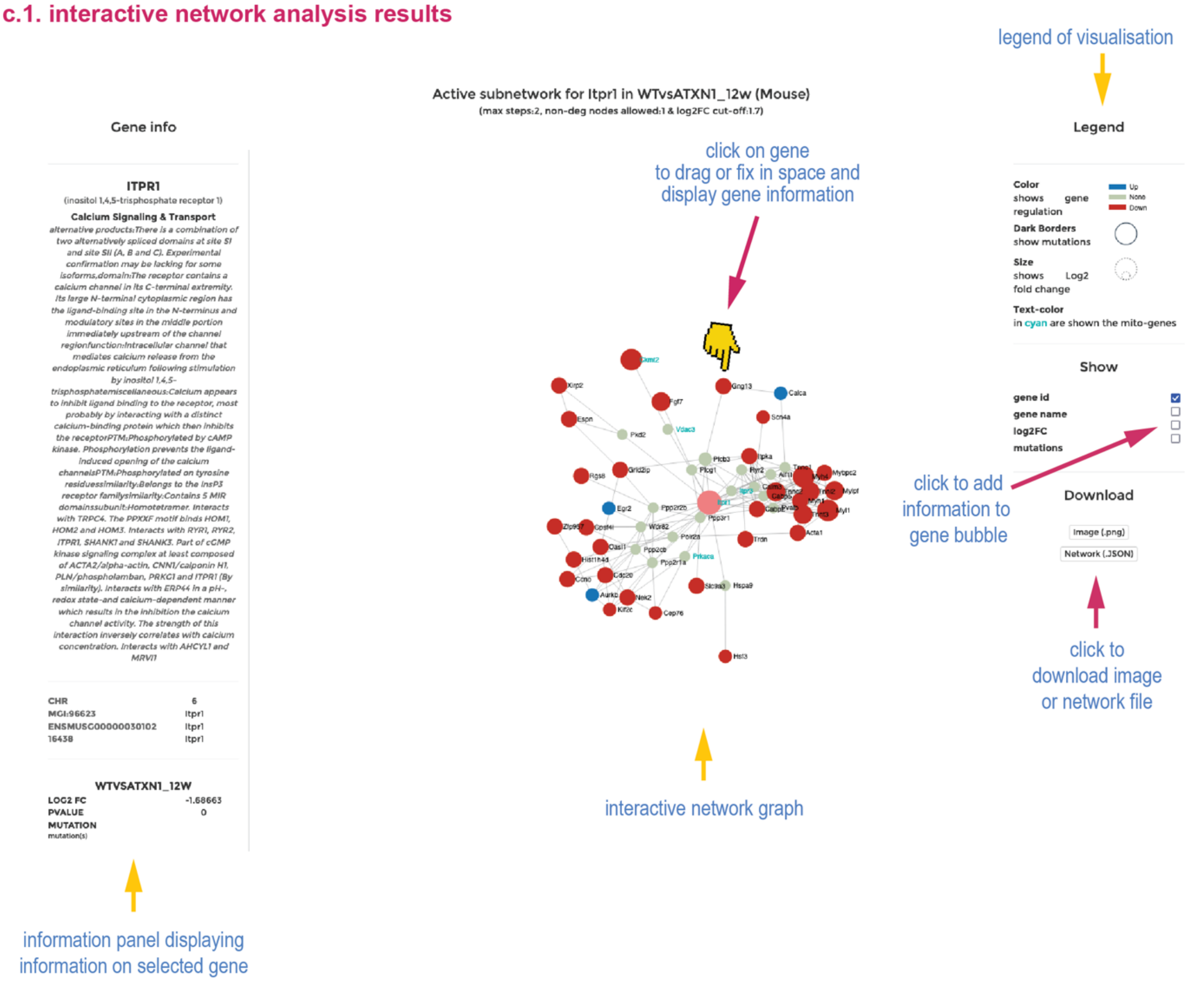

References

1. Letourneau,A., Santoni,F.A., Bonilla,X., Sailani,M.R., Gonzalez,D., Kind,J., Chevalier,C., Thurman,R., Sandstrom,R.S., Hibaoui,Y., *et al.* (2014) Domains of genome-wide gene expression dysregulation in Down's syndrome. *Nature*, **508**, 345–350.

2. Liu,Y., Borel,C., Li,L., Müller,T., Williams,E.G., Germain,P.-L., Buljan,M., Sajic,T., Boersema,P.J., Shao,W., *et al.* (2017) Systematic proteome and proteostasis profiling in human Trisomy 21 fibroblast cells. *Nat Commun*, **8**, 1212.

3. Yim,A., Koti,P., Bonnard,A., Marchiano,F., Dürrbaum,M., Garcia-Perez,C., Villaveces,J., Gamal,S., Cardone,G., Perocchi,F., *et al.* (2020) mitoXplorer, a visual data mining platform to systematically analyze and visualize mitochondrial expression dynamics and mutations. *Nucleic Acids Res.*, **48**, 605–632.

4. Meiler,A., Marchiano,F., Haering,M., Weitkunat,M., Schnorrer,F. and Habermann,B.H. (2021) AnnoMiner is a new web-tool to integrate epigenetics, transcription factor occupancy and transcriptomics data to predict transcriptional regulators. *Sci Rep*, **11**, 15463–21.
